# Computational virology: From the inside out^☆^

**DOI:** 10.1016/j.bbamem.2016.02.007

**Published:** 2016-07

**Authors:** Tyler Reddy, Mark S.P. Sansom

**Affiliations:** Department of Biochemistry, University of Oxford, South Parks Road, Oxford OX1 3QU, UK

**Keywords:** Virus, Envelope, Membrane, Simulation, Molecular dynamics, Coarse grained

## Abstract

Viruses typically pack their genetic material within a protein capsid. Enveloped viruses also have an outer membrane made up of a lipid bilayer and membrane-spanning glycoproteins. X-ray diffraction and cryoelectron microscopy provide high resolution static views of viral structure. Molecular dynamics (MD) simulations may be used to provide dynamic insights into the structures of viruses and their components. There have been a number of simulations of viral capsids and (in some cases) of the inner core of RNA or DNA packaged within them. These simulations have generally focussed on the structural integrity and stability of the capsid and/or on the influence of the nucleic acid core on capsid stability. More recently there have been a number of simulation studies of enveloped viruses, including HIV-1, influenza A, and dengue virus. These have addressed the dynamic behaviour of the capsid, the matrix, and/or of the outer envelope. Analysis of the dynamics of the lipid bilayer components of the envelopes of influenza A and of dengue virus reveals a degree of biophysical robustness, which may contribute to the stability of virus particles in different environments. Significant computational challenges need to be addressed to aid simulation of complex viruses and their membranes, including the need to integrate structural data from a range of sources to enable us to move towards simulations of intact virions.

This article is part of a Special Issue entitled: Membrane Proteins edited by J.C. Gumbart and Sergei Noskov.

## Highlights

• Molecular simulations provide dynamic insights into the structures of viruses.
• Simulations of viral capsids have probed their structural stability.
• Simulations of HIV-1, influenza, and dengue virus envelopes are described.
• Computational challenges in simulations of complex viral membranes are discussed.

## Graphical abstract

**Figure:**
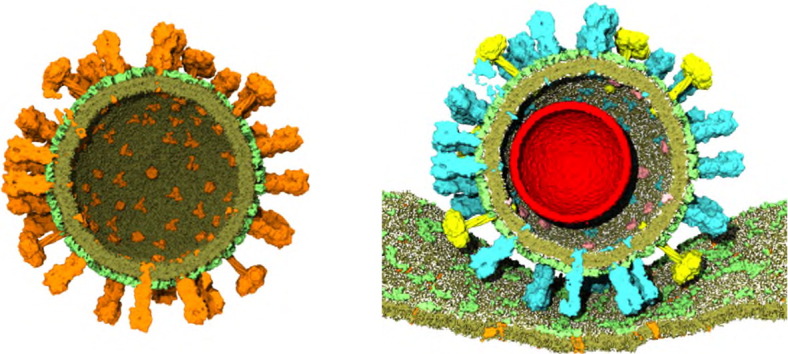

## 1 Introduction

Viruses cause a plethora of human illnesses, resulting in > 1.5 million annual deaths worldwide (WHO Fact sheet 310). Viruses also indirectly regulate the carbon flux of our planet [1], they are used to attack cancer cells (oncolytic viruses; reviewed elsewhere [2]), and are leveraged for a number of biotechnology applications (e.g. virus-like particles, VLPs, decorated with tumour-associated carbohydrate antigens as anti-cancer vaccines [3]; and the packaging of enzymes within VLPs [4]). At a more fundamental level they help us to probe many aspects of the biology of cells, including the organization and dynamics of cell membranes.

**Fig. 1:**
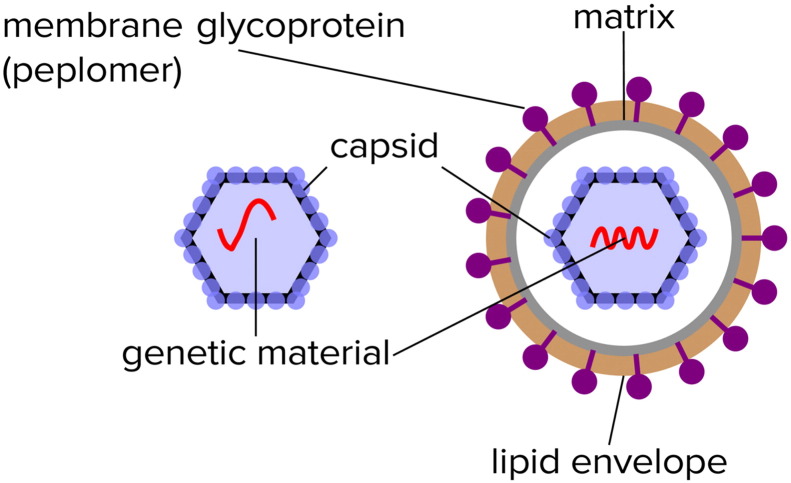
Non-enveloped and enveloped viruses: a simple schematic diagram illustrating the relationship between viral capsids and viral envelopes, and emphasizing that the envelope is made up of lipids and transmembrane glycoproteins (peplomers).

There has been considerable progress over the past two decades in the structural biology of viruses, employing X-ray crystallography, and cryoelectron microscopy and tomography. Viruses may be classified as non-enveloped (in which case the genome is surrounded by a protein capsid) and enveloped (in which case the capsid or nucleoprotein core is surrounded by a viral membrane envelope, containing both proteins and lipids; Fig. 1). Structural studies have provided many high resolution structures of capsids, and also structures of envelopes, the latter often determined by cryoelectron microscopy and tomography [5]. Viral envelopes are derived from the membranes of the host cell. Thus, studies of the organization of viral envelopes may also provide insights into the organization and dynamics of cell membranes [6].

Molecular dynamics (MD) simulations, both atomistic (AT) and coarse-grained (CG), have been widely used to probe the dynamics and organization of cell membranes and their proteins [7], [8]. In the context of viruses, molecular simulations may be used to e.g. probe the conformational dynamics of viral capsid proteins [9], to explore the organization and stability of viral capsids [10], and to aid in the modelling and interpretation of the structure of viral envelopes. In this review we will focus on large scale simulations of viral capsids and envelopes, looking towards simulations of intact virions, and conclude with the prospect of large scale simulations of virion/cell membrane and virion/antibody interactions. We will not cover viral membrane fusion with host cells, as this has been discussed elsewhere (e.g. [11]). In particular we will consider how the nature of the capsid (in non-enveloped viruses) or of protein–lipid interactions within the envelope of a virion may aid us in assessing the biophysical stability of virions, some of which are capable of extended survival in fluctuating environments.

We will structure our discussion from the inside of the virus outwards, starting from simulations of the inner core and capsid of selected viruses, and progressing out towards recent simulation studies of viral envelopes. A summary of the major simulation studies reviewed is provided in Table 1.

## 2 The inner core

We start with the genetic material inside of the virion. There are fewer structural details available for this region and hence simulations are less well developed than for the outer surface. Simulating a complete model of the genetic material inside a virion may provide crucial insights into the viral assembly process/nucleation of components, and ultimately may also lead to improved methods for engineered packaging of therapeutics inside capsids. For example, brome mosaic virus capsid proteins can assemble into different-sized capsids to accommodate nanoparticles of different sizes [12], [13], [14], [15]. Cargo size-driven variation in icosahedral capsid morphology has been replicated in coarse-grained computational models [16]. For increasing genome sizes, the entropic cost of polymer confinement into a roughly spherical capsid can rise without limit [17].

It has been suggested that the assembly of small ssRNA viruses is primarily driven by electrostatic interactions between the RNA and basic residues in the protein, which are often concentrated in arginine-rich-motifs (ARMs) in the carboxy and/or amino terminal regions of the proteins [18]. When RNA strands substantially exceed the length of the native genome, multiple capsids connected by a strand of RNA can share the packaging burden [19]. A survey of experimental data indicates that capsid ARMs only partially offset the negative charge of packaged RNA [20]. In a recent study [21], it was shown that overcharging (nucleic acid negative charge > capsid protein positive charge) is favourable from kinetic and thermodynamic standpoints. A complex dependence of the optimal genome length on capsid charge, capsid size, RNA structure (extent of base pairing) and excluded volume is revealed.

**Fig. 2:**
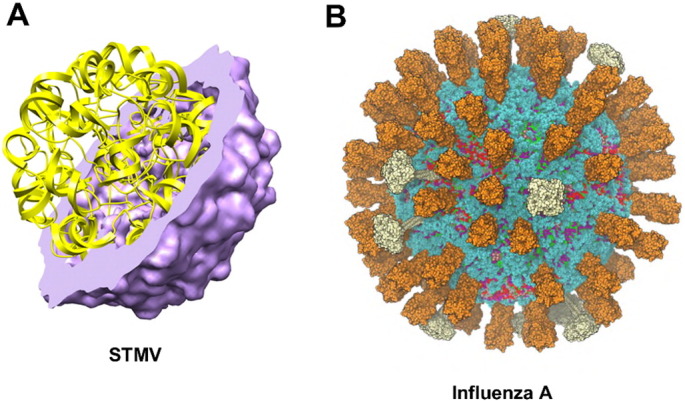
Examples of (A) the viral capsid (purple) plus ssRNA genome (yellow) of STMV ([23]; figure courtesy of S.C. Harvey) modelled at atomic resolution; and (B) the viral envelope membrane of influenza A [82] modelled at coarse-grained resolution.

An approximation of the RNA genome structure from STMV was simulated in isolation and found to be substantially more stable than the isolated outer protein shell of the virion in the absence of RNA [22]. Stability (i.e., of overall radius and of RMSD) was conferred, despite an artificial nucleotide sequence and lack of tertiary structure interactions, suggesting that the RNA core may have a substantial role in assembly and maintenance of a stable virion. The STMV model has been extended to include all of the atoms of the virion, including the natural genomic sequence with a likely tertiary structure [23] (Fig. 2A), although this more complete model has not (yet) been explored in extended molecular dynamics simulations. Likewise, Pariacoto virus (PaV) genomic RNA has been modelled to match experimental electron densities, although experimental details are only available for ~ 35% of the ultrastructure [24]. The remaining 65% was modelled by an expansion, coarse-grained minimization, and shrinking procedure followed by re-mapping to all-atom coordinates. Two separate all-atom models were generated, and the electrostatic stability and consistency with experimental observations was greatest for a PaV model in which polycationic capsid protein tails penetrated deeply into the RNA core. This study also suggests that the RNA core may play a role in driving nucleation and assembly of the virion, and is compounded by the lack of experimental observations of empty PaV capsids. A largely electrostatic role for RNA in capsid assembly has also been suggested by Langevin dynamics simulations of PaV [25]. Monte Carlo simulations of CG RNA in the Cowpea Chlorotic Mottle Virus capsid also suggest strong interactions between the genome and the positively-charged N-terminal tails of the capsid proteins [26]. Despite stabilization with 92 structural Ca^2 +^ ions, in the absence of RNA the satellite tobacco necrosis virus (STNV) capsid expanded over the course of two microsecond-length simulations at different salt concentrations [27], again supporting the proposed role of RNA in virion structural integrity. However, the combination of charged self-repulsion of ssRNA and steric interaction with the rugged inner topography of the capsid protein in bacteriophage MS2 CG simulations suggests that the capsid is also capable of shaping the outer shell of genomic RNA [28]. The relative paucity of high-resolution structural information for viral genomes may be dealt with in simulation contexts by using chloride ions to probe the probable location of the negatively charged genomes inside the capsid [29]. This strategy may eventually be used to guide the construction of more accurate computational models of virions in cases where only partial genomic structural data are available. However, a number of experiments indicate that interactions between capsid protein and RNA are sequence-specific [30], [31], [32], [33]. Indeed, genomic stem-loops that nucleate capsid assembly have recently been described for STNV and two RNA phages [34], [35]. Thus it may be important to include these effects in models and simulations of virions. Stem-loop based viral capsid assembly mechanisms were recently reviewed along with a proposed assembly mechanism for those capsids that do not depend on stem-loops for capsid protein binding [36].

Simulations of many DNA-containing capsids are complicated by the frequent use of motor-driven genome packaging and by large pressure differentials. The ~ 50 nm persistence length of dsDNA and its high charge density are thought likely to preclude spontaneous incorporation to the capsid (discussed in [37]). Indeed, whilst viral packaging motors are the strongest of all biological motors, their mechanism of action remains controversial [38]. CG simulations have been used to model dsDNA-containing capsids, addressing the thermodynamics and kinetics of motor-driven insertion, as recently reviewed [39]. Both single-stranded and double-stranded DNA segments have been simulated in a model of the Adeno-Associated Viral capsid [40] in which the structure of the capsid was simplified to a smooth sphere and the genome was represented with a 6:1 nucleotide:CG particle mapping scheme. In general, CG simulations of DNA insertion push DNA pseudoatoms into the capsid one-by-one, with an equilibration after each packaging step. For example, DNA packaging in bacteriophage P4 was studied using MD equilibration at 0.3 K after each pseudoatom was inserted to reveal that a concentric spool was the optimal packaged genome structure [41]. A thermodynamic analysis of MD simulations for packaging of DNA into bacteriophage epsilon15 predicted a requisite force of 125 pN to overcome electrostatic and entropic contributions [42]. Brownian dynamic simulations were used to determine that bacteriophage DNA loading force increases more than 10-fold during the final third of the packaging process [43]. Langevin dynamics have been used to simulate the packing process of a ds-DNA bacteriophage [44], and suggest that the packing process may be stochastic rather than deterministic in character.

There have also been a number of studies characterizing the reverse process, i.e. release of the genome from the viral capsid. Mesoscale simulations were used to determine that for semiflexible polymers such as DNA, a sphere packs and ejects more rapidly than an ellipsoid, suggesting that roughly-spherical phages may have evolved to optimize the ejection process [45]. Simulations also suggest that DNA ejection can be efficiently controlled by tuning the salt concentration of the environment [46]. From a kinetic standpoint, Langevin dynamics simulations indicate that DNA ejection speed is maximal at an intermediate stage of ejection (i.e., rather than at the beginning of the process) [47]. Conversely, another set of Langevin dynamic simulations of ds-DNA ejection from a bacteriophage suggested that early stage ejection rates were the highest [48]. Stochastic simulation techniques have shown that delocalized knots in a packaged bacteriophage genome do not interfere with the ejection process [49].

## 3 The capsid

Following the first atomic-resolution structure of a virus [50], there have been a number of simulations of viral capsids (Table 1), largely focussing on aspects of capsid stability. A number of studies have probed the structural integrity of viral capsids by force probe MD (as a theoretical complement to AFM studies of virion integrity) [51], [52]. These simulations did not include the genomic material (see above) despite the potential structural importance of the viral genome. Long timescale computational assessments of the stability of a range of viral capsids have been performed using highly (~ 200 atoms:1 CG particle) coarse grained MD simulations [10]. The capsids of three plant satellite viruses (STMV, satellite panicum mosaic virus (SPMV), and STNV) all collapsed over the course of simulations ranging from 5 to 25 μs when a model of the genomic viral RNA was absent. However, the capsid of brome mosaic virus (BMV), the bacteriophage ϕX174 procapsid, the reovirus core, and the poliovirus capsid were all stable in the absence of genetic material in CG simulations ranging between 1.5 and 11 μs in duration. BMV capsid collapse was observed after 5 μs of simulation when the N-terminal protein tails were removed, consistent with experimental evidence that cleavage is a pre-requisite for capsid disassembly and the release of virulence factors into the host cell [53]. Thus, simulation approaches appear to replicate and predict experimental results relating to the stability of viral capsids and the structural role played by the packaged genome. In the future, cryo-EM may play an increasingly important role in identifying the most appropriate genomic RNA secondary structures proposed by computational approaches, as was recently done for STMV [54].

Whilst it should be clear from above that the structural integrity of many virions depends on mutual interactions between genetic material and the surrounding capsid, there are a number of capsid proteins which form stable native structures in vitro in the absence of genetic material. Some of these capsid proteins have been studied extensively from a computational standpoint in an effort to understand the fundamentals of self-assembly (reviewed by [37], [55]). The thermodynamics of capsid self-assembly may be considered similar to those of surfactant micelle formation [56], with hydrophobic interactions between capsomers representing the primary driving force for self-assembly. Conversely, electrostatic interactions between protein subunits can oppose capsid assembly, and hepatitis B virus capsid stability increased with ionic strength [57]. Capsids exhibit critical concentrations of asymmetric units analogous to critical micelle concentrations. The high concentrations of asymmetric units required for these simulations necessitate a high degree of coarse graining. However, a number of simplifications may be acceptable given that assembly behaviour is the same for explicit solvent and Brownian dynamics simulations [37]. State-based approaches do not track diffusive motions of capsid subunits and enable access to larger systems/longer timescales, but unlike particle-based approaches they have not been able to identify malformed capsid traps. CG particle-based simulations have been described in three classes [37]: patchy-sphere models (spherical excluded volume with short-range interaction sites), a second model [58] with multiple pseudoatoms and short-range interaction sites, and a third class of models with polygonal interaction directions, no diffusion tracking, and direct placement of subunits into growing capsids [59], [60].

The first reported usage of MD to study viral capsid self-assembly employed simple triangular shaped subunits with only two types of spherical particles: those that define the shape/space occupancy, and those that define permitted interaction sites with other subunits [61]. Self-assembly of 1000 subunits into 15 pentakisdodecahedral shells was observed over the course of ~ 0.5 million integration steps. However, this simplified proof-of-concept study did not correspond to a specific virus structure nor was it possible to estimate a time scale for the process. This work was later extended with more sophisticated trapezoidal capsomers for larger capsid structures [62]. In the latter simulations it was necessary to impose nonphysical restrictions to prevent the formation of spurious non-icosahedral structures. An alternative CG approach also used a highly simplified representation of capsomers [63], and employed discontinuous molecular dynamics (DMD), where all interactions are of the square-well type and solvent is treated implicitly. The formation of full capsids was optimal at a capsomer concentration of 90 μM and temperature of ~ 310 K, and in these simulations it was also possible to identify mis-assembled structures (partial capsids and ‘monster’ particles). The final addition step of a capsomer to the nearly-formed capsid was found to be rate-limiting. A Brownian dynamics computational study using a single spherical excluded volume per capsomer, with internal bond vectors to represent protein shape and complementarity, found that partially assembled intermediates can interact during the assembly process [64]. In general, intermediates do not build up during capsid assembly—the proteins are almost entirely in either the free subunit configuration or in the fully-assembled capsid (reviewed by Hagan [37]). Optimal assembly is described for the cases where subunit–subunit interactions are reversible.

These capsid self-assembly simulations are highly coarse-grained, and their timescales are therefore difficult to estimate. The hepatitis B virus capsid assembly has been experimentally observed on the order of ~ 10 min [65], and capsid proteins generally assemble on time scales varying from seconds to hours (discussed in [37]). Icosahedral capsid assembly may be described by two timescales—nucleation and elongation [66]. Simulated critical nuclei have ranged in size between 3 and 10 subunits [16], [67], [68], [69]. Hagan [37] notes that although the critical nucleus should correspond to a half capsid in most equilibrium cases, equilibrium is effectively never achieved on experimental or simulation timescales. In addition, the majority of icosahedral capsid simulations currently do not account for allosteric effects. However, it is clear that efforts to simulate full-scale enveloped virions (see below) could benefit from more complete models of the (nucleo)capsid. It is likely that the resolution gap between all-atom/moderately coarse grained whole virion simulations and highly coarse grained capsid self-assembly simulations may be bridged by hybrid multiscale models [70].

## 4 Enveloped viruses

There have been fewer simulation studies of enveloped viruses, reflecting their greater structural complexity, the lack of symmetry in the bilayer and/or the envelope, and their generally larger size, all of which necessitate more complex model building prior to simulation. These aspects demand very large scale computational resources and/or multiscale approaches to their simulation.

### 4.1 Capsid

Recently, the mature HIV-1 native capsid structure was determined by a combination of cryo-EM and all-atom molecular dynamics simulations [71]. A 100 ns duration simulation of the 64 million atom HIV-1 capsid was performed in explicit solvent and demonstrated the importance of capsid protein pentamers at crucial locations in the structure. The immature HIV-1 capsid protein has also been represented in a moderately coarse-grained model that successfully reproduced the hexameric lattice in a 100 ns MD simulation [72]. Similarly, coarse-grained capsid protein self-assembly simulations demonstrated the importance of the trimer of dimers fundamental structural unit in the HIV-1 capsid self-assembly process [73]. Furthermore, the first all-atom model of an immature retroviral lattice was recently produced [74], with the simulated RSV Gag lattice model demonstrating the structural importance of regions upstream and downstream of the capsid protein within the Gag polyprotein. A complete atomic model of the RHDV capsid is also available, thanks to a mixture of structural biology and simulation techniques [75]. These studies extend those of capsids of non-enveloped viruses, and provide key structural elements for incorporation into complete virion models and simulations.

### 4.2 Matrix

**Fig. 3:**
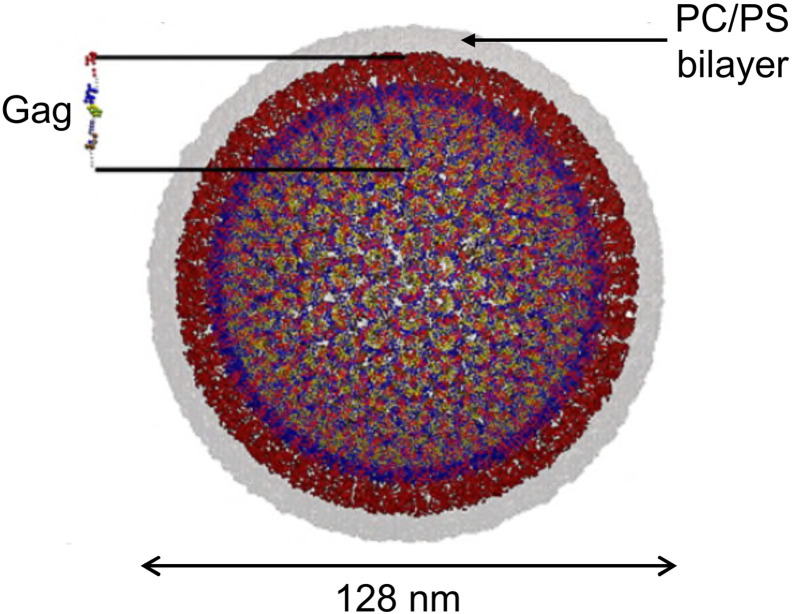
A coarse grained model of the immature HIV-1 virion ([72]; figure courtesy of G. Voth) showing the surrounding PS/PC lipid bilayer (135,802 lipid molecules) in grey and the 2034 Gag proteins (matrix, MA, domain in red) immediately inside this.

There is often a layer of matrix protein located between the capsid or ribonucleoprotein core of enveloped virions and the membrane envelope proper. This is true for paramyxoviruses, orthomyxoviruses, herpesviruses (the tegument), retroviruses, and filoviruses [76]. In a landmark study, a coarse-grained model of the immature HIV-1 Gag protein (which contains the matrix domain; see Fig. 3) was simulated in a model of the complete immature virion for 100 ns [72]. In this model the lipid bilayer contained both zwitterionic (PC) and anionic (PS) lipids which surrounded the matrix domains of the (uncleaved) Gag proteins. A recent CG simulation study of the HIV-1 matrix protein suggests that it interacts with and recruits PIP_2_ molecules in membrane domains [77]. Given recent advances in simulations of bilayers containing complex mixtures of lipids, including PIP_2_ [78], [79], [80], it therefore should be possible to explore matrix/lipid interactions in an intact model of an immature HIV-1 virion in more detail.

These studies demonstrate the importance of more complete models of matrix protein assemblies than are generally available in helping to establish the role of interactions between matrix proteins and the envelope bilayer in viral organization and assembly. Although high resolution structures of individual matrix proteins or their domains have been determined (e.g. M1 from influenza A, PDB ID 1AA7, for which there is a simple model of its likely membrane in the OPM database http://opm.phar.umich.edu/ [81]), high-resolution structures of viral matrix protein assemblies are often not available or remain controversial. Simulations may aid in modelling matrix assembly configurations to be used in whole virion models.

### 4.3 Envelope

We will now review simulation studies of the outside layer of enveloped viruses. We will illustrate such simulations via a recent study of the influenza A virus [82] (see Fig. 2B), and also provide a brief comparison with recent simulations of dengue virus [83].

As noted above for HIV-1 Gag, specific lipid/protein interactions may play a role in maintaining virion integrity. Although one may estimate the lipid composition of an enveloped virion based on the lipid composition of its host cell source membrane, there is considerable evidence that viruses select unique lipid compositions for their protective envelopes. Thus, knowledge of a viral lipidome is a key piece of information for the construction of a computational model of an enveloped virion. Viral lipidomes are currently available (at various degrees of resolution) for: influenza A [84], [85], HIV [86], Hepatitis C [87], human cytomegalovirus [88], vesicular stomatitis virus [89], Sindbis virus [90], Semliki Forest virus [91], Simian virus 5 (canine parainfluenza virus) [92], Rous sarcoma virus + Newcastle disease virus + Sendai virus [93], frog virus 3 (inner membrane only) [94], Chilo Iridescent Virus [95], the two forms of *Autographa californica* nuclear polyhedrosis virus [96], vaccinia virus [6], murine leukaemia virus [97], herpes simplex virus [98], and bacteriophage PM2 [99]. Indeed, vaccinia virus can even be deactivated with detergent and reactivated by incorporation of specific lipids (especially PS) [100], and these highly complex poxviruses may have up to three protective lipid envelopes [101]. The lipidome for Ebola virus has not yet been determined [102], although importantly it has been suggested that VP40, the most abundant protein of the Ebola virus, can interact with anionic lipids of mammalian cell membranes to form filamentous virus-like particles. Thus, it should in principle be possible to simulate viral envelope bilayers for a number of different viruses.

The other key elements for enveloped virus simulations are the membrane proteins embedded within the lipid bilayer. Although a number of structures of the extra-membrane domains of these proteins have been determined, in most cases complete structures of the proteins including their TM domains are not available. Thus a modelling approach based on TM domain prediction and/or structural data is required. This is analogous to the situation for a number of mammalian membrane receptors such receptor tyrosine kinases, where model TM structures have been used to e.g. probe lipid/protein interactions [103], [104]. The supporting matrix and nucleocapsid ultrastructures often remain controversial, and in silico construction of a full-scale viral envelope is methodologically challenging (see below).

**Fig. 4:**
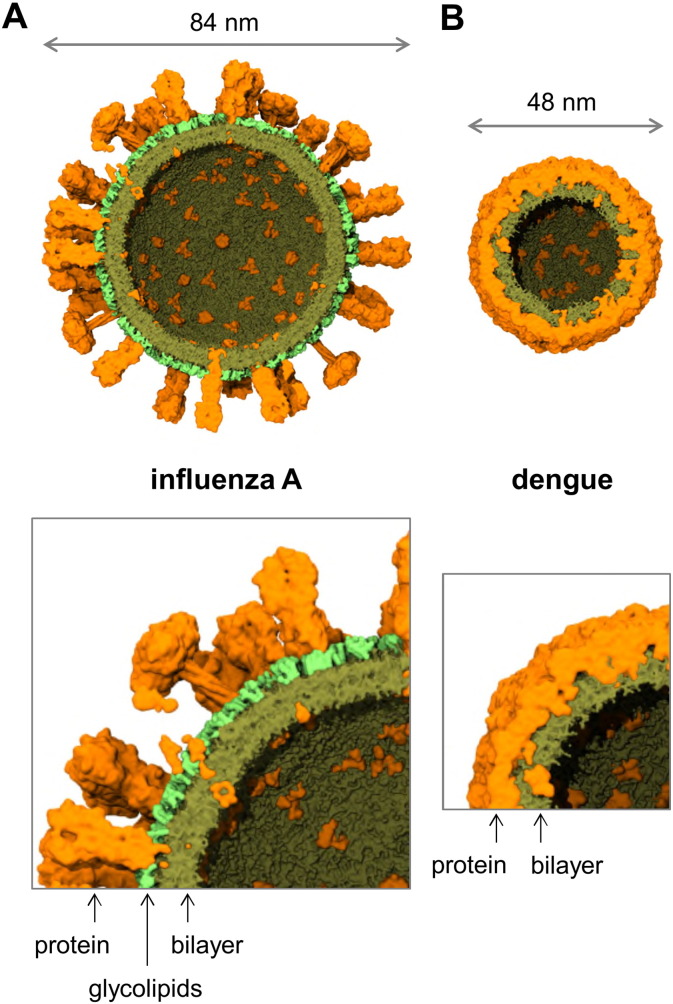
Comparison of coarse-grained models of the envelopes of (A) influenza A [82] and (B) dengue virus [83]. In each case a cross-section of the entire virion envelope model is shown, along with a zoomed in section below. Protein (orange) and lipid (dark green) components of the envelope are shown, with the glycolipid headgroups of the influenza A envelope shown in bright green.

Despite these challenges, it is possible to model and simulate complete virion envelopes. This has recently been possible for the spherical form of the influenza A virion, combining crystal structures and TM domain models for the HA and NA proteins, an NMR structure for the TM domain of the M2 protein, and a reasonable approximation to the experimentally determined lipidome (Figs. 2B & 4A). In this study [82] the M1 matrix protein was modelled indirectly by restraining the mobility of the transmembrane glycoproteins which likely interact with the M1 layer below the bilayer of the membrane envelope.

The outer diameter and sphericity (shape) of the simulated influenza A virions were both stable for 5 μs, consistent with the demonstrated stability of the virus in aqueous solution [105]. The prevalence of glycans on the surface of the outer leaflet of the lipid bilayer of the influenza A model suggested that antibody or therapeutic compound access to the M2 proton channel may have to overcome steric barriers. The three species of influenza A envelope protein translated only modestly over the surface of the cholesterol-rich membrane (i.e., they did not individually explore a large fraction of the available surface), with diffusion constants matching previous experimental measurements by solid-state NMR [106]. Despite the much larger ectodomains of HA and NA, the smaller M2 protein diffused more slowly in the bilayer, perhaps reflecting a larger cross-sectional membrane area and stronger interactions with surrounding lipids. The inter-peplomer spacing (i.e. the spacing between membrane glycoproteins) on the influenza A surface was consistent with previous experimental measurements [107], and thus amenable to bivalent antibody binding. These spacings could also be probed in the context of multivalence, suggesting that polyvalent interactions between HA and/or NA on the viral surface and sialic acid residues on the host membrane are likely. This would enable strong virus-host association despite relatively weak (~ 2–3 mM affinity) viral HA-single host receptor interaction in vitro [108].

**Fig. 5:**
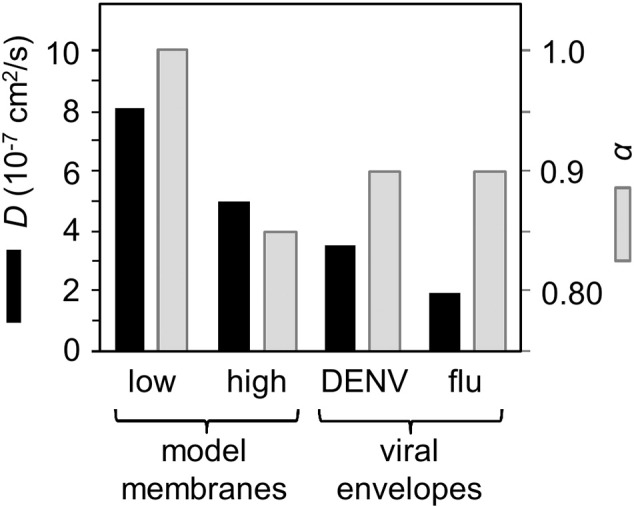
Comparison of lipid diffusion coefficients for: a simple model membrane [111] containing a low or a high fraction of the membrane surface area occupied by protein; dengue virus [83]; and influenza A [82]. The black bars correspond to the values of the diffusion coefficient, the grey bars to the values of the scaling exponent α.

It would be of considerable interest if recent HIV-1 whole-virion envelope simulations [72] could be extended beyond 100 ns to enable comparison of overall ultrastructure stability, diffusive behaviour, and inter-protein spacing with the influenza A results. Both HIV-1 and influenza A virion envelope simulations to date have exhibited properties consistent with experimental measurements. We have also been able to probe diffusive properties of lipids within the outer envelope of the dengue virion (Fig. 4B; [83]). The lack of cholesterol in the dengue virion envelope was apparently counterbalanced by the dense crowding of protein transmembrane domains and the near-complete enclosure of the outer leaflet of the lipid bilayer with a protein shell. This resulted in lipid diffusive properties similar to those in the raft-like influenza A virion membrane, namely reduced diffusion coefficients *D* and exponents α less than 1 (Fig. 5), the latter indicative of anomalous diffusion. All three sets of whole virion envelope simulations (i.e. HIV-1, influenza A, and dengue) provide potential platforms for probing virion environmental stability and interactions (i.e., with antibodies, therapeutics, etc.). Furthermore, some cell membranes (i.e., erythrocytes [109], [110]) are naturally cholesterol-rich and crowded with proteins, and could be explored by similar computational approaches to those used to probe virions. Previous simulation studies on crowded bacterial membrane models [111] exhibited diffusive behaviour similar to the virions discussed above (Fig. 5).

Thus, simulation of full-scale virions may enable us to assess basic stability and the dynamics of antibody binding between proteins on the surface. In particular, we can determine if protein spacing on the viral surface is compatible with simultaneous binding of each Fab region of a Y-shaped antibody to neighbouring viral proteins. These details may not be fully captured in isolated simulations of e.g. viral peplomers [112], or in simulations in which symmetry is imposed on an isolated asymmetric unit. Furthermore, protein-lipid interactions on the surface may be crucial for assessing the biophysical stability of enveloped virions.

### 4.4 Simulation considerations

As noted above, there are considerable computational challenges both in building and in simulating models of complete virion envelopes. Packmol [113] was used extensively in the early stages of building a complete coarse-grained model of the human influenza A virion [82]. Packmol depends on knowledge of a relatively-straightforward geometry based scripting language. More recent developments include autoPACK and cellPACK which together provide an infrastructure to automatically integrate experimental structural data from various sources in order to build mesoscale (10–100 nm) models of heterogeneous and complex biological assemblies. This approach has been used to generate mesoscale models of both the HIV-1 virion and of synaptic vesicles [114]. It may prove possible to use such models as starting configurations for higher resolution (CG and AT) modelling. LipidWrapper is a promising choice for generating a lipid bilayer configuration of arbitrary overall geometry that is ready for atomistic or coarse-grained MD simulation [115], as the tiling strategy used within this method is less susceptible to steric conflicts than the progressive incorporation strategy used in various other approaches.

When a complex membrane assembly on the scale of an intact virion (i.e. ~ 100 nm) has already been constructed at near-atomic resolution, it is often desirable to perform ‘computational mutagenesis,’ replacing or modifying lipids and proteins that are already present in the initial model. A procedure was recently described for incorporation of new components into a system by progressively increasing the radii and electrostatic interaction strengths of nascent particles (i.e. an ‘alchemical’ procedure) [116], to avoid traditional problems with steric conflicts in model manipulation. For example, this latter approach was used to transmute sphingomyelin to the Forssman glycolipid in our computational model of the influenza A virion envelope [82]. We recently employed Alchembed to relax steric conflicts in a computational model of the dengue virion (Fig. 4B), where lipids were propagated from an equilibrated asymmetric unit simulation to the whole virion using icosahedral symmetry operators. Both Alchembed and g_membed [117] are well suited to virion construction procedures where proteins are to be inserted into lipid bilayers. The former is capable of embedding multiple proteins at arbitrary angles, whilst the latter may require a measure of scripting to deal with multiple insertions at different angles. In both cases, however, it can be highly non-trivial to select the appropriate locations for protein insertion within the viral structure. A common strategy described for the construction of models of both retroviruses and the influenza A virus is the placement of charged particles on a spherical surface followed by the evolution of Coulombic repulsion to achieve a roughly equidistant distribution of seed points for protein placement [82], [118].

**Fig. 6:**
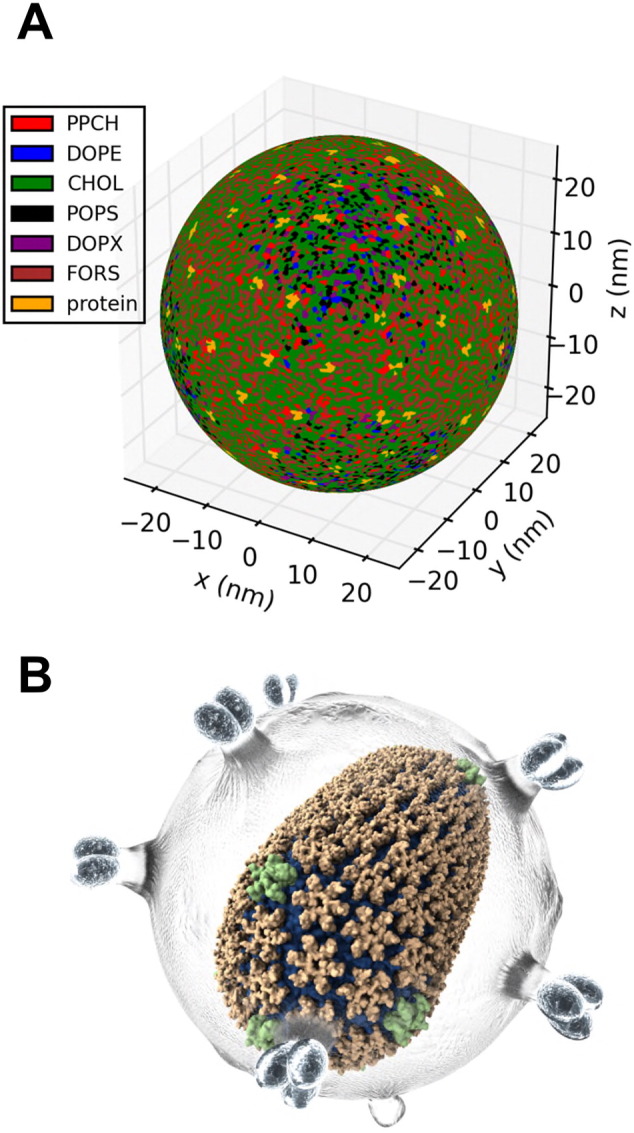
(A) Spherical Voronoi diagram for the outer leaflet of a model of the influenza A envelope [82]. Lipid molecules within the leaflet are coloured as follows: POPS (palmitoyl oleoyl phosphatidylserine; black), PPCH (hydroxylated palmitoyl sphingomyelin with an ethanolamine headgroup; red), DOPE (di-oleoyl phosphatidylethanolamine; blue), CHOL (cholesterol; green), DOPX (ether-linked DOPE; purple), FORS (Forssman glycolipid; brown). Protein is shown in yellow. (B) Illustration of the use of Quicksurf to provide a VMD rendering of the mature HIV capsid (orange and green) within a conceptual rendering of the viral envelope (grey) ([118]; figure courtesy of J.R. Perilla).

Computational virology simulations can range from a few million particles [82] to hundreds of millions of particles [71], which can necessitate extensive benchmarking to evaluate scaling of simulations on supercomputer resources exploiting thousands of computer cores and associated GPUs. It is often necessary to tune the distribution of tasks to CPUs and GPUs and to regulate the communication between nodes on a cluster to achieve maximal performance [119]. Given that many viruses are spherical or have other regular 3-dimensional shapes, it is appropriate to leverage techniques from the field of computational geometry [120] to perform biophysical analyses. For example, calculation of the surface area, volume and shape (sphericity) of a virion depend on calculation of the convex hull: effectively a convex polyhedron enclosing the full set of particles of the virion. We have exploited this to accurately calculate the area per molecule of lipids and (transmembrane) proteins in the envelopes of virions using a spherical Voronoi diagram (Fig. 6A). The code for this (https://github.com/scipy/scipy/pull/5232) is currently under review for incorporation into the well-established scipy library [121].

Large scale simulations of virions also present challenges for visualization. The need for GPU-accelerated visualization tools such as the “Quicksurf” and Optix ray tracing utilities in VMD [122] has recently been described in detail for computational studies of retroviral capsids [118] (Fig. 6B). In our work we have used these facilities of VMD for visualization, and in addition have made extensive use of the open-source MDAnalysis [123], numpy/scipy [121], and IPython/Jupyter [124] libraries to analyse large viral simulation systems using multiple computer cores. GPU-accelerated hyperball representations may also become useful for interactive models of massive viral systems [125]. Computational virology requires us to exploit multicore computer architectures at the simulation, analysis, and visualization stages of a project.

## 5 Future challenges

**Fig. 7:**
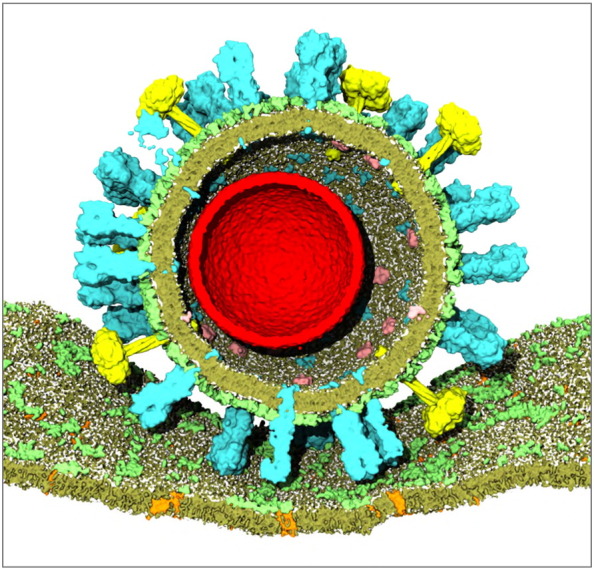
Model of an influenza A virion (with the red sphere indicating the approximate location of the genome within the virion, not currently modelled) [82] docked against a simple model of a mammalian cell membrane with glycolipids in pale green, other lipids in darker green, and cell membrane proteins in orange.

There are a number of clear challenges to future computational studies of enveloped viruses. Glycosylation of viral surface proteins can influence antigenicity [126], and a key enhancement to future full-scale virion simulations will be the incorporation of models of glycans on these proteins. For extended time scale coarse grain simulations it may be a challenge to reconcile the inherent flexibility of glycans [127] with the elastic networks that are used to maintain protein tertiary structure. Heterogeneity, even at a single site of glycosylation (i.e., protein glycoforms [128]), will also be difficult to capture in silico. Simulations of virions interacting with antibodies should be possible in the near future, whilst simulations of virions binding to models of target cell membranes (Fig. 7) are likely to require methodological innovation. Extending adaptive multiscale models (i.e., those used for HIV-1 envelope/Gag simulations [72]) to facilitate incorporation of data from multiple and orthogonal experimental approaches would also be a welcome route towards reducing the extensive manual intervention currently required in building full-scale virion models for simulations.

## Transparency document

Transparency document

## Figures and Tables

**Table 1:** Summary of selected MD simulations of viruses.

Virus	Components	Diameter (nm)	Granularity & duration	Reference
*Non-enveloped viruses*				
STMV	Capsid + RNA	17	AT; 13 ns	[22], [23]
		CG^1^; 5 μs	[10]
PaV	Capsid + RNA	20	AT; 0*	[24]
STNV	Capsid	20	AT; 1 μs	[27]
		CG^1^; 7 μs	[10]
SBMV	Capsid	36	AT; 0.1 μs	[10]
SPMV	Capsid	17	CG^1^; 25 μs	[10]
BMV	Capsid	28	CG^1^; 5 μs	[10]
Poliovirus	Capsid	33	CG^1^; 11 μs	[10]
Reovirus	Protein core	75	CG^1^; 1.5 μs	[10]

*Enveloped viruses*				
Influenza A	Envelope proteins + lipids	84	CG^2^; 5 μs	[82]
Dengue virus	Envelope proteins + lipids	48	CG^2^; 5 μs	[83]
HIV-1	Mature capsid	~ 120 × 60	AT; 0.1 μs	[71]
Immature virion envelope: protein + lipids	125	CG^3^; 0.1 μs	[72]

Abbreviations: STMV (satellite tobacco mosaic virus), PaV (pariacoto virus), STNV (satellite tobacco necrosis virus), SBMV (southern bean mosaic virus), SPMV (panicum mosaic satellite virus), BMV (brome mosaic virus).

* = energy minimization only.

CG^1^ = ~ 1:200 atom to CG mapping; CG^2^ = ~ 1:4 atom to CG mapping; CG^3^ = a hybrid multiscale CG approach was used.
